# Individual, Sociodemographic, and Environmental Factors Related to Physical Activity During the Spring 2020 COVID-19 Lockdown

**DOI:** 10.3389/fpsyg.2021.643109

**Published:** 2021-03-16

**Authors:** Claudia Teran-Escobar, Cyril Forestier, Clément Ginoux, Sandrine Isoard-Gautheur, Philippe Sarrazin, Anna Clavel, Aïna Chalabaev

**Affiliations:** ^1^Laboratoire SENS, Univ. Grenoble Alpes, Grenoble, France; ^2^Laboratoire PACTE, Univ. Grenoble Alpes, Grenoble, France; ^3^Laboratoire Motricité, Interactions, Performance, MIP - EA4334, Le Mans Université, Le Mans, France

**Keywords:** physical activity, COVID-19 pandemic, psychology, context, exercise

## Abstract

**Background:** Research has shown important between-individual variations in physical activity (PA) during the COVID-19 lockdown.

**Objectives:** The objectives of this is study are to examine the individual, sociodemographic, and environmental factors related to PA during the spring 2020 COVID-19 lockdown in France and to explore the mediating and moderating role of intention and self-efficacy toward PA in the relationships between sociodemographic/environmental variables and PA.

**Design:** In this cross-sectional study, participants living in France (*N* = 386) completed an online survey between March 30 and April 10, 2020.

**Method:** Minutes per week of moderate-to-vigorous PA during the lockdown; usual physical activity before the lockdown; and psychological (e.g., intention, self-efficacy, and autonomous and controlled motivation), sociodemographic (gender, age, and number of children), and environmental (habitat surface area and type of housing) factors were measured in the survey. Multiple linear regressions were used to investigate the role of these predictors on PA. Intention and self-efficacy were also examined as moderators and mediators of the association between sociodemographic/environmental factors and PA.

**Results:** Usual physical activity before the COVID-19 lockdown, intention toward PA, habitat surface area, and controlled motivation significantly predicted PA during the lockdown. No mediating effects of intention or self-efficacy were found. Intention significantly moderated the association between gender and PA and the association between part-time work and PA.

**Conclusions:** PA during the COVID-19 lockdown was mainly predicted by individual factors and notably usual PA. These results highlight the important role of habits in a highly changing context.

## Introduction

COVID-19 represents one of the most important sanitary crises in the last decades. Beyond the effects of COVID-19 on physical health, the disease may also have side effects on mental health, due to the strategies most countries have adopted for restraining contagion (e.g., lockdowns and closure of restaurants, schools, and public places) and other related events (e.g., overload media exposure of COVID-19, Garfin et al., 2020). In France for example, the government has implemented a national lockdown from 17th March to 11th May, 2020. People were authorized to go out of their home only for necessity reasons (work, medical check-up, purchases of necessities, or physical activity for <1 h per day and <1 km from home) and with a signed certificate. In the work domain, the lockdown has generated important differences between workers. While teleworking became the rule for most companies, workers who were unable to work (e.g., hotel industry and construction) were either placed in partial unemployment (and continued receiving around 85% of their salaries) or lost their jobs in the case of precarious contracts (e.g., interim workers and nannies).

To limit the side effects of these restrictive measures, the WHO (2020), researchers (Chen et al., 2020), and local and national governments published a series of recommendations so that people could remain sufficiently physically active (i.e., by giving advices about how to maintain physical activity during the lockdown). Indeed, regular physical activity is known as an important source of physical health (e.g., benefits for the immune system, Nieman and Wentz, 2019) and mental well-being (e.g., reduced depression and anxiety symptoms, Rebar et al., 2015). For example, being physically active has been associated with greater well-being during the spring 2020 COVID-19 lockdown (Green et al., 2020; Lesser and Nienhuis, 2020; Qin et al., 2020; Schuch et al., 2020; Ginoux et al., 2021). In contrast, physical inactivity and sedentary behaviors have been associated with increased stress and anxiety (Meyer et al., 2020).

Despite WHO recommendations and the potential benefits of physical activity during COVID-19 lockdown, a reduction of physical activity from 7 to 38% in European countries has been observed during the week of March 22 (FitBit, 2020). Although some activities (e.g., walking for commuting) have decreased while other activities (e.g., working out indoors) have increased (Cheval et al., 2020; Garmin, 2020), a recent literature review of 41 articles indicates that physical activity has globally decreased during lockdown worldwide (Caputo and Reichert, 2020). However, this review also indicates significant between-individual variations in the impact of lockdown on physical activity: while some people managed to remain sufficiently active during lockdown, others were mostly inactive. It is therefore essential to identify the factors of physical activity during this period in order to better adapt physical activity recommendations during physical and social isolation.

The sociocognitive approach has been dominant to examine factors of physical activity (for a review, see Rhodes et al., 2019). Sociocognitive theories (e.g., Theory of Planned Behavior, Ajzen, 1991; Health Belief Model, Rosenstock, 1974) consider that behavior depends on reasoned cognitions: people act when they have formed the intention to do so, which emerges when they believe they are capable to perform the behavior (e.g., self-efficacy), and that the behavior has consequences that are more positive than negative (e.g., perceived benefits and risks). Another prominent approach is the self-determination theory (Ryan and Deci, 2017), which considers behavior to depend on motivations that are either internal to the individual—when behavior is the result of a personal choice and act of volition (i.e., autonomous motivation)—or external to the individual—when behavior results from perceived internal or external pressure (i.e., controlled motivation).

Although psychological theories are useful to explain engagement in physical activity, they have mostly focused on psychological factors and have omitted the role of external ones. Yet, there is evidence that sociodemographic and environmental factors also substantially predict physical activity. For example, research in different countries showed a tendency of women to be less physically active than men (for a review, see Guthold et al., 2018). Other research has shown an inverse association between age and physical activity, with younger people being more physically active than older people (e.g., Bauman et al., 2012). Moreover, physical activity has been associated with employment status and family type, with people working in full-time jobs and having children being less physically active (e.g., Rhodes et al., 2014; Borodulin et al., 2016). Past research has also shown that people in higher socioeconomic positions might be more active during leisure time than people in lower socioeconomic ones (Gidlow et al., 2006; Beenackers et al., 2012) and have more home equipment for leisure-time physical activity (Cerin and Leslie, 2008). In contrast, people in lower socioeconomic positions seem to be more active during work (Beenackers et al., 2012).

Other studies have identified environmental factors on physical activity, including walkability, housing type, access to open spaces/recreation facilities, aesthetic variables (e.g., places evaluated as attractive), and mixed land use (coexistence of shops, residences, and other buildings in the same neighborhood/zone) (for a review, see Durand et al., 2011; Bauman et al., 2012).

In sum, it is necessary to consider not only psychological factors but also external ones to better understand physical activity participation. This integrative approach is particularly relevant in the context of the COVID-19 crisis, which has caused sudden changes in people's work, family, and living environment.

Based on the aforementioned literature, we investigated individual-level factors, including psychological (i.e., intention, self-efficacy, autonomous, and controlled motivation, as well as factors that may be particularly relevant in this sanitary crisis situation, such as perceived risks of being contaminated, perceived stress, and vitality) and behavioral (i.e., usual physical activity before the lockdown) factors, as well as sociodemographic (i.e., age, gender, education, employment, household, and socioeconomic status) and environmental (i.e., type of housing, habitat surface areas, region's degree of COVID-19 contamination, access to sports equipment, and the media exposure) factors.

A recent study conducted at the same time as the present research suggests that individual-level factors predict more physical activity than environmental ones (Rhodes et al., 2020). This study indeed observed that the main predictors of physical activity during lockdown were exercise identity and extraversion. Only one environmental factor, sports equipment at home, significantly predicted physical activity. The present study also examined this question and went a step further, by investigating how individual and external factors articulate with each other. Several studies suggest that external variables (e.g., sociodemographic and environmental) may influence behavior through the mediating role of social cognitions (e.g., intention and self-efficacy) (Cerin and Leslie, 2008; Sniehotta et al., 2013; Hagger and Hamilton, 2020). In contrast, other studies (e.g., Sniehotta et al., 2013; Schüz et al., 2019) suggest that sociocognitive constructs interact with sociodemographic/environmental variables to predict physical activity. For instance, Sniehotta et al. (2013) showed that the relationships between social cognitions and physical activity were stronger for individuals with better physical health and lower levels of socioeconomic deprivation. Moreover, Schüz et al. (2019) observed that more educated people presented a stronger relationship between intention and physical activity.

To investigate the relationships between individual and external factors and moderate-to-vigorous physical activity during the COVID-19 lockdown, we adopted the same model comparison approach as in Sniehotta et al. (2013), by investigating the three following competing hypotheses:

Hypothesis 1. Sociodemographic (i.e., age, gender, number of children, employment status, and educational attainment), environmental (i.e., type of housing, habitat surface area, access to sports equipment, and media exposure), and individual (e.g., usual physical activity before COVID-19 lockdown, intention, self-efficacy, autonomous motivation, controlled motivation, subjective vitality, stress, and perceived risks of getting COVID-19) variables predict physical activity during the COVID-19 lockdown independently from each other.
Hypothesis 2. The relationships between environmental/sociodemographic variables and physical activity during the lockdown are mediated by intention and self-efficacy.
Hypothesis 3. The relationships between environmental/sociodemographic variables and physical activity during the lockdown are moderated by intention and self-efficacy.

## Materials and Methods

### Participants and Procedure

An *a priori* power analysis conducted using G. Power 3.1.9.4 (Faul et al., 2007; Erdfelder et al., 2009) indicated that 308 participants were needed, considering 47 predictors (21 single predictors and 26 interactions), an *R*^2^ of 0.40 (based on similar research, Sniehotta et al., 2013), and 90% power. Participants aged 18 and over and residing in France were recruited to answer an online survey (about 20 min). Recruitment was done using social media (i.e., Facebook and Twitter) and by word of mouth. To encourage participation, our research laboratory committed to donating 0.50€ to bioclinical research on COVID-19 for each completely fulfilled questionnaire. The survey was available between March 30 (2 weeks after the French government announced the lockdown) and April 10, 2020.

Three-hundred-and-eighty-six people (65.54% women; *M*_age_ = 33.09, SD = 13.18) completed the survey, after reading and signing an online informed consent form.

### Measures

Physical activity during lockdown was assessed based on the International Physical Activity Questionnaire (IPAQ, Craig et al., 2003), which was adapted to better reflect the extraordinary circumstances of COVID-19 lockdown. Participants reported the time in minutes on different physical activity categories. These categories were chosen based on a recent opinion article about how to maintain physical activity levels during COVID-19 (Chen et al., 2020). Participants were also asked to add the time spent doing any other physical activities and, in this case, to define these activities. We then classified each activity into moderate-to-vigorous physical activity when it was superior or equal to 3 METS (metabolic equivalent task, which is the amount of energy that is used during an activity) using the compendium of physical activities of Ainsworth et al. (2011).

Usual physical activity before the lockdown was assessed using the Saltin–Grimby Physical Activity Questionnaire (Grimby et al., 2015).

Intention to do physical activity was assessed using one item from Godin (2012), and self-efficacy related to physical activity was assessed using one item (Schwarzer et al., 2015). Autonomous and controlled motivation toward physical activity was assessed using a short version of the “motivation scale toward health-oriented physical activity” (Boiché et al., 2019). The eight items reflected four motivational regulations: intrinsic, identified, introjected, and external regulations. Intrinsic and identified regulations were averaged to obtain autonomous motivation, and introjected and external regulations were averaged to obtain controlled motivation (Brunet et al., 2015). Autonomous motivation showed good reliability (α = 0.89). However, because controlled motivation did not show good reliability (α = 0.55), we decided to remove one item. Reliability after removing this item was acceptable (α = 0.61). Subjective vitality was assessed using the Subjective Vitality Scale (Ryan and Frederick, 1997), showing good reliability (α = 0.90), and perceived stress was assessed using a French translation of the short form of Perceived Stress Scale (PSS-4, Warttig et al., 2013), showing good reliability (α = 0.81). Finally, the perceived risks of getting coronavirus were assessed using perceived susceptibility and perceived severity scales. Perceived susceptibility of getting coronavirus disease was adapted from a scale related to the susceptibility of getting influenza infection (Nexøe et al., 1999). This scale did not show good reliability (α = 0.48). Therefore, we decided not to include it in our analyses. Perceived severity of coronavirus disease was assessed and adapted from perceived severity scale of getting influenza infection (Nexøe et al., 1999). Reliability was good for this scale (α = 0.77).

Media exposure was assessed to gather information about the extent to which the search of information has or has not increased since the start of lockdown. Four items measured four different sources of information (e.g., television, Internet, social networks, and press). Reliability was acceptable for this scale (α = 0.64) (more details of the scales in Supplementary Material).

Sociodemographic information included age, gender, number of children, employment status (full-time work, partial-time work, partial unemployment, or no job), educational attainment, type of housing (housing with access to green areas or terrace and housing without access to green areas or terrace), habitat surface area, region's degree of contamination (regions most affected by coronavirus were classified as red, regions less affected as yellow, and the regions the least affected as green), and access to sports equipment at home (yes or no).

### Analytical Procedures

Moderate-to-vigorous physical activity (MVPA) did not have a normal distribution, and squared root transformation was applied to approximate a normal curve. Once MVPA was transformed, skewness and kurtosis were examined to check for normality.

All hypotheses were tested using multiple linear regressions in R version 3.6.0. The “Lm” function was used to test the first and second hypotheses, and “olsrr” package (Hebbali, 2020) was used to do stepwise regression analyses (Hypothesis 3). Dummy variables were created for the categorical variables (gender, employment status, type of housing, and access to sports equipment at home).

Hypothesis 1 was tested using hierarchical regression analyses. In the first step, all the sociodemographic and environmental variables were included as predictors. In the second step, individual variables (intention, self-efficacy, autonomous motivation, controlled motivation, subjective vitality, perceived stress, perceived severity of COVID-19, and usual physical activity before lockdown) were additionally included following the methodology used by Sniehotta et al. (2013). Finally, both models were compared using chi-square difference tests to decide which model better explained behavior.

Hypothesis 2 was investigated using mediation analysis following recommendations of Yzerbyt et al. (2018), which showed that the joint significance test has a better balance of type I error and statistical power, compared to other approaches such as the bias-corrected bootstrap method. Intention and self-efficacy were tested as mediators between all other variables (environmental, sociodemographic, and individual) and physical activity. In the first step, we tested whether sociodemographic, environmental, and individual variables (except intention and self-efficacy that were tested as hypothesized mediators) predicted physical activity. In the second step, we tested whether sociodemographic, environmental, and individual (except the hypothesized mediators) variables predicted each of the hypothesized mediators (intention and self-efficacy). In the third step, we tested whether each mediator predicted physical activity when controlling for sociodemographic, environmental, and individual variables. According to the joint significance method, an indirect effect is claimed when regression coefficients in the second and third steps are significant.

Hypothesis 3 was tested using stepwise forward regression analyses. Stepwise forward regression is a method that selects and retains predictors based on mathematical criteria (e.g., Akaike information criterion), the final model containing the best predictors of the outcome and the best fitting indices (Field et al., 2012). In the first step, we centered all predictors using subtract mean to avoid multicollinearity problems (e.g., Shieh, 2011; Iacobucci et al., 2016). In the second step, physical activity was regressed on all sociodemographic, environmental, and individual variables. In the third step, interactions between sociodemographic and environmental variables, on the one hand, and intention and self-efficacy, on the other hand, were included. Finally, significant interactions were decomposed into simple slope analyses and Johnson-Newman plots using the package “interactions” (Long, 2019). To simplify these analyses, all the variables were scaled using the scale function in R (this function subtracts the mean and divides each value by the standard deviation).

After testing each hypothesis, we followed recommendations to assess the independence of residuals (using Durbin–Watson test), normal distribution of residuals (using bar plot and q–q plot), and non-multicollinearity (using VIF function in “car” package, Fox and Weisberg, 2019).

## Results

### Descriptive Statistics

The sample population reported performing an average of 368 min of moderate-to-vigorous physical activity (MVPA) per week (SD = 251.12). The means, standard deviations, and the description of our variables are presented in Table 1. Correlations between variables are displayed in Supplementary Material.

**Table 1:** Means, standard deviations, and description of variables.

**Variable**	**Mean (95% CI)**	**SD**	**Range/unity of measure**
Dependent variable			
PA during COVID-19 lockdown	368 (342.74, 393.34)	251.12	Minutes per week
Sociodemographic and environmental Variables			
Gender	65.54% women and 34.46% men		
Age	33.09 (31.76, 34.41)	13.18	
Region classified by color (green zones are the least affected by COVID-19, red zones are the most affected zones)	63% people living in yellow zones, 19.2% people living in green zones, and 17.9% people in red zones		
Educational attainment	6.04 (5.92, 6.16)	1.20	0–7
Employment status	45.08% full-time job, 32.9% no work, 12.7% part-time job, and 9.3% partial unemployment		
Type of housing	68.65% access to green spaces/balcony and 31.35 % without access to green spaces/balcony		
Habitat surface area	99.41 (94.37, 104.45)	49.88	Square meters
Number of Children	0.55 (0.46, 0.64)	0.91	
Media exposure	5.52 (5.35, 5.68)	1.65	1–10
Access to sports equipment at home	69.69% access to sports equipment and 32.9% without access to sports equipment		
Psychological and individual variables			
Intention	5.60 (5.43, 5.77)	1.67	1–7
Self-efficacy	5.27 (5.1, 5.45)	1.76	1–7
Autonomous motivation	5.62 (5.5, 5.74)	1.20	1–7
Controlled motivation	1.84 (1.76, 1.93)	0.87	1–7
Subjective vitality	4.31 (4.18, 4.44)	1.30	1–7
Perceived stress	3.60 (3.55, 3.66)	0.55	1–7
Perceived severity of getting COVID	2.9 (2.74, 3.04)	1.48	1–7
Usual physical activity before lockdown	3.03 (2.94, 3.12)	0.90	1–4

*N = 387. PA, physical activity; CI, confidence interval; SD, standard deviation. Values between parentheses represent confidence intervals*.

### Did Sociodemographic/Environmental and Individual Factors Independently Predict Physical Activity (Hypothesis 1)?

Hierarchical multiple linear regression analyses were performed to test Hypothesis 1 (see Table 2). The first model including all sociodemographic and environmental variables was significant [*F*_(12, 354)_ = 4.27, *p* < 0.001], with an *R*^2^ of 0.13. Gender (*β* = 0.11^*^, *p* = 0.040), habitat surface area (*β* = 0.13^*^, *p* = 0.035), and not having access to sports equipment at home (*β* = −0.24^***^, *p* < 0.001) were significantly associated with physical activity.

**Table 2:** Hierarchical regression models testing the independent contribution of sociodemographic, environmental, and individual variables to physical activity during COVID-19 lockdown (Hypothesis 1).

	**Model 1**				**Model 2**			
	***b***	**SE *b***	***β***	***p***	***b***	**SE *b***	***β***	***P***
Constant	15.84^***^	2.90		**<0.001**	−5.67	3.86		0.14
	(10.13, 21.54)				(−13.27, 1.93)			
Gender	1.57^*^	0.76	0.11^*^	**0.040**	0.27	0.67	0.02	0.688
	(0.08, 3.06)				(−1.05, 1.59)			
Age	−0.01	0.03	−0.02	0.669	−0.001	0.03	−0.002	0.967
	(−0.07, 0.04)				(−0.05, 0.05)			
Region degree of contamination	0.49	0.58	0.04	0.397	0.46	0.49	0.04	0.348
	(−0.65, 1.62)				(−0.50, 1.41)			
Educational attainment	0.03	0.35	0.01	0.937	0.27	0.30	0.05	0.361
	(−0.67, 0.72)				(−0.31, 0.86)			
Part-time job	−0.48	1.18	−0.02	0.686	−0.10	1.01	−0.01	0.919
	(−2.79, 1.84)				(−2.09, 1.88)			
Partial unemployment	0.51	1.27	0.02	0.686	0.15	1.10	0.01	0.891
	(−1.99, 3.02)				(−2.01, 2.32)			
No job	1.33	0.97	0.09	0.170	1.33	0.82	0.09	0.105
	(−0.57, 3.23)				(−0.28, 2.94)			
Housing without access to green areas/terrace	−0.15	0.87	−0.01	0.863	1.14	0.75	0.08	0.130
	(−1.87, 1.57)				(−0.34, 2.62)			
Habitat surface area	0.02^*^	0.01	0.13^*^	**0.034**	0.02^*^	0.01	0.11^*^	**0.037**
	(0.001, 0.04)				(0.001, 0.03)			
Number of children	−0.59	0.41	−0.08	0.157	−0.33	0.36	−0.04	0.358
	(−1.40, 0.23)				(−1.03, 0.37)			
No access to sports equipment	–3.68^***^	0.78	−0.24^***^	**<0.001**	−0.90	0.71	−0.06	0.203
	(−5.21, −2.15)				(−2.30, 0.49)			
Media exposure	0.02	0.21	0.01	0.917	0.31^t^	0.18	0.07^t^	0.092
	(−0.40, 0.44)				(−0.05, 0.67)			
Intention					0.99^***^	0.26	0.24^***^	**<0.001**
					(0.47, 1.51)			
Self-efficacy					0.36	0.27	0.09	0.174
					(−0.16, 0.86)			
Autonomous motivation					0.17	0.31	0.03	0.595
					(−0.45, 0.79)			
Controlled motivation					–0.72^*^	0.36	−0.09^*^	**0.048**
					(−1.44, −0.01)			
Subjective vitality					0.50^t^	0.27	0.09^t^	0.068
					(−0.04, 1.04)			
Perceived stress					0.35	0.58	0.03	0.545
					(−0.79, 1.48)			
Perceived severity					−0.22	0.21	−0.05	0.299
					(−0.63, 0.20)			
Usual physical activity before lockdown					2.49^***^	0.43	0.32^***^	**<0.001**
					(1.66, 3.33)			
***R*** ^**2**^	**0.13**				**0.40**			
**Adjusted** ***R***^**2**^	**0.10**				**0.36**			

*N_Model 1_ = 367, N_Model 2_ = 351. Dependent variable is minutes of moderate-to-vigorous physical activity per week transformed in squared root. Women were used as reference dummy group; results in this table are displayed for men. b, raw coefficient; SE b, standard error of betas, *β*, standardized betas*.

t *p < 0.10*,

* *p < 0.05*,

*** *p < 0.001. Values between parentheses represent confidence intervals. Bold values are significant (p < 0.05)*.

The second model, which included individual variables in addition to sociodemographic/environmental ones, was significant [*F*_(20, 330)_ = 10.95, *p* < 0.001] with an *R*^2^ of 0.40. Usual physical activity before the lockdown (*β* = 0.32^***^, *p* < 0.001), intention (*β* = 0.24^***^, *p* < 0.001), habitat surface area (*β* = 0.11^*^, *p* = 0.037), and controlled motivation (*β* = −0.09^*^, *p* = 0.048) were significantly associated with physical activity during COVID-19 lockdown. Durbin–Watson test (Durbin and Watson, 1971) (Durbin–Watson_Model 1_ = 1.84, Durbin–Watson_Model 2_ = 1.97), quantile–quantile plot (available in Supplementary Material), as well as VIF tests (Mansfield and Helms, 1982) (average VIF_Model 1_ = 1.18, average VIF_Model 2_ = 1.24) suggested that residuals were normally distributed and not autocorrelated (i.e., Durbin–Watson values should be between 1.5 and 2.5; Field et al., 2012 and VIF values should not be bigger than 10; Field et al., 2012). Finally, the chi-squared tests showed that the second model (the extended one) better explained physical activity than the first model.

### Did Psychological Factors (Intention and Self-Efficacy) Mediate the Association Between Sociodemographic/Environmental Factors and Physical Activity (Hypothesis 2)?

The first multiple regression of the mediation analysis (see Table 3, model 3) tested whether sociodemographic/environmental and individual variables (excluding intention and self-efficacy) predicted physical activity. This regression was significant [*F*_(18, 332)_ = 9.19, *p* < 0.001] with an *R*^2^ of 0.33. Usual physical activity before the lockdown (*β* = 0.38^***^, *p* < 0.001), subjective vitality (*β* = 0.15^**^, *p* = 0.003), autonomous motivation (*β* = 0.13^*^, *p* = 0.015), and controlled motivation (*β* = −0.10^*^, *p* = 0.042) were significant predictors. Durbin–Watson_Model 3_ = 1.95 and average VIF_Model 3_ = 1.18.

**Table 3:** Hierarchical regression models testing the mediating role of intention and self-efficacy in the association between sociodemographic/environmental variables and physical activity during COVID-19 lockdown (Hypothesis 2).

	**Model 3**				**Model 3.1**				**Model 3.2**			
	***β***	**SE *b***	***β***	***p***	***b***	**SE *b***	***β***	***p***	***β***	**SE *b***	***β***	***p***
Constant	−2.34	4.01		0.560	2.76^**^	1.01		0.007^**^	1.44	1.00		0.151
	(−10.23, 5.55)				(0.78, 4.74)				(−0.53, 3.42)			
Gender	0.15	0.71	0.01	0.827	−0.10	0.18	−0.03	0.573	−0.03	0.18	−0.01	0.887
	(−1.23, 1.54)				(−0.45, 0.25)				(−0.37, 0.32)			
Age	−0.01	0.03	−0.02	0.683	−0.01	0.01	−0.07	0.188	−0.00	0.01	−0.03	0.583
	(−0.07, 0.04)				(−0.02, 0.01)				(−0.02, 0.01)			
Region degree of contamination	0.53	0.51	0.05	0.300	0.05	0.13	0.02	0.712	0.07	0.13	0.03	0.565
	(−0.48, 1.54)				(−0.21, 0.30)				(−0.18, 0.33)			
Educational attainment	0.30	0.31	0.05	0.342	0.03	0.08	0.03	0.658	−0.04	0.08	−0.03	0.619
	(−0.32, 0.92)				(−0.12, 0.19)				(−0.19, 0.12)			
Part-time job	0.11	1.06	−0.01	0.919	0.21	0.26	0.04	0.435	0.10	0.26	0.02	0.702
	(−1.98, 2.19)				(−0.31, 0.73)				(−0.28, 0.86)			
Partial unemployment	0.14	1.15	0.01	0.904	−0.12	0.29	−0.02	0.676	0.29	0.29	0.05	0.319
	(−2.13, 2.41)				(−0.69, 0.45)				(−0.28, 0.57)			
No job	1.26	0.86	0.09	0.142	−0.13	0.22	−0.04	0.539	0.15	0.21	0.04	0.499
	(−0.43, 2.95)				(−0.56, 0.29)				(−0.28, 0.57)			
Housing without access to green areas/terrace	1.14	0.79	0.08	0.148	0.03	0.20	0.01	0.863	−0.07	0.20	−0.02	0.717
	(−0.41, 2.69)				(−0.36, 0.43)				(−0.46, 0.32)			
Habitat surface area	0.01^t^	0.01	0.10^t^	0.090	−0.002	0.002	−0.06	0.291	0.00	0.00	−0.01	0.934
	(−0.002, 0.03)				(−0.01, 0.002)				(−0.00, 0.00)			
Number of children	−0.46	0.37	−0.06	0.220	−0.10	0.09	−0.05	0.309	−0.12	0.09	−0.06	0.209
	(−1.19, 0.28)				(−0.28, 0.09)				(−0.30, 0.07)			
No access to sports equipment	−0.99	0.74	−0.07	0.186	−0.07	0.19	−0.02	0.709	−0.04	0.19	−0.01	0.813
	(−2.45, 0.48)				(−0.44, 0.30)				(−0.41, 0.32)			
Media exposure	0.29	0.19	0.07	0.134	−0.02	0.05	−0.02	0.732	0.01	0.05	0.01	0.913
	(−0.09, 0.67)				(−0.11, 0.08)				(−0.09, 0.10)			
Autonomous motivation	0.76^*^	0.31	0.13^*^	**0.015**	0.45^***^	0.08	0.33^***^	**<0.001**	0.41^***^	0.08	0.29^***^	**<0.001**
	(0.15, 1.38)				(0.29, 0.60)				(0.26, 0.57)			
Controlled motivation	–0.77^*^	0.38	−0.10^*^	**0.042**	0.01	0.10	0.01	0.901	−0.16^t^	0.10	−0.08^t^	0.095
	(−1.52, −0.03)				(−0.18, 0.20)				(−0.35, 0.03)			
Subjective vitality	0.82^**^	0.27	0.15^**^	**0.003**	0.18^*^	0.07	0.14^*^	**0.011**	0.41^***^	0.07	0.30^***^	**<0.001**
	(0.28, 1.36)				(0.04, 0.31)				(0.27, 0.54)			
Perceived stress	−0.07	0.60	−0.01	0.912	–0.31^*^	0.15	−0.10^*^	**0.041**	−0.27^t^	0.15	−0.08^t^	0.077
	(−1.25, 1.11)				(−0.61, −0.01)				(−0.56, 0.03)			
Perceived severity	−0.14	0.22	−0.03	0.527	0.06	0.06	0.05	0.296	0.06	0.06	0.05	0.292
	(−0.57, 0.29)				(−0.05, 0.17)				(−0.05, 0.17)			
Usual physical activity before lockdown	2.94^***^	0.44	0.38^***^	**<0.001**	0.32^**^	0.11	0.17^**^	**0.004**	0.36^**^	0.11	0.19^**^	**0.001**
	(2.07, 3.80)				(0.10, 0.54)				(0.15, 0.58)			
***R*** ^**2**^	**0.33**				**0.24**				**0.34**			
**Adjusted** ***R***^**2**^	**0.30**				**0.20**				**0.30**			

*N_Model 3_ = 351, N_Model 3.1_ = 353, N_Model 3.2_ = 353. In model 3, dependent variable is minutes of moderate-to-vigorous physical activity per week transformed in squared root. In model 3.1, dependent variable is intention, and in model 3.2, dependent variable is self-efficacy. Women were used as reference dummy group; results in this table are displayed for men. b, raw coefficient; SE b, standard error of betas, *β*, standardized betas*.

t *p < 0.10*,

* *p < 0.05*,

** *p < 0.01*,

*** *p < 0.001. Values between parentheses represent confidence intervals. Bold values are significant (p < 0.05)*.

Second, in model 3.1 (Table 3), intention was regressed on the same predictors used in model 3. The regression was significant [*F*_(18, 334)_ = 5.97, *p* < 0.001] with an *R*^2^ of 0.24. Autonomous motivation (*β* = 0.33^***^, *p* < 0.001), usual physical activity before lockdown (*β* = 0.17^**^, *p* = 0.004), subjective vitality (*β* = 0.14^*^, *p* = 0.011), and perceived stress (*β* = −0.10^*^, *p* = 0.041) were significantly associated with intention to do physical activity. Durbin–Watson_Model 3.1_ = 2.01 and average VIF_Model 3.1_ = 1.18.

In model 3.2 (Table 3), self-efficacy was regressed on the same predictors. This model was significant [*F*_(18, 334)_ = 9.52, *p* < 0.001] with an *R*^2^ of 0.34. Subjective vitality (*β* = 0.30^***^, *p* < 0.001), autonomous motivation (*β* = 0.29^***^, *p* < 0.001), and usual physical activity before lockdown (*β* = 0.19^**^, *p* = 0.001) were significantly related to self-efficacy to do physical activity. Durbin–Watson_Model 3.2_ = 2.06 and average VIF_Model 3.2_ = 1.19.

We decided to stop the mediation analyses at this stage because there was no sociodemographic or environmental factor that was significantly associated to both physical activity and one of the potential mediators (intention or self-efficacy).

### Did Psychological Factors (Intention and Self-Efficacy) Interact With Sociodemographic/Environmental Factors in the Prediction of Physical Activity (Hypothesis 3)?

Given the high number of predictors when adding interactive terms, a stepwise forward multiple regression analysis was performed to test Hypothesis 3. The final model is detailed in Table 4. This model was significant [*F*_(29, 321)_ = 8.64, *p* < 0.001] with an *R*^2^ of 0.44. Usual physical activity before the lockdown (*β* = 0.28^***^, *p* < 0.001), intention (*β* = 0.20^*^, *p* = 0.022), media exposure (*β* = 0.10^*^, *p* = 0.031), and controlled motivation (*β* = −0.11^*^, *p* = 0.020) were significantly related to physical activity.

**Table 4:** Stepwise regression model testing interaction effects between intention, self-efficacy, and sociodemographic/environmental variables on physical activity during COVID-19 lockdown (Hypothesis 3).

	**Model 4**			
	***b***	**SE *b***	***β***	***P***
Constant	17.46^***^	0.63		**<0.001**
	(16.21, 18.70)			
Usual physical activity before lockdown	2.20^***^	0.43	0.28^***^	**<0.001**
	(1.36, 3.04)			
Self-efficacy	0.14	0.31	0.04	0.652
	(−0.48, 0.76)			
Habit surface area	0.02^t^	0.01	0.10t	0.053
	(0.00, 0.03)			
Controlled motivation	–0.85^*^	0.36	−0.11^*^	**0.020**
	(−1.52, −0.11)			
Subjective vitality	0.47^t^	0.27	0.09^t^	0.085
	(−0.07, 1.01)			
Part-time job	0.19	1.00	−0.01	0.852
	(−1.79, 2.16)			
Partial unemployment	0.28	1.09	0.01	0.794
	(−1.86, 2.43)			
No job	1.26	0.81	0.09	0.122
	(−0.34, 2.85)			
Media exposure	0.40^*^	0.18	0.10^*^	**0.031**
	(0.04, 0.76)			
Gender	0.23	0.66	0.02	0.725
	(−1.07, 1.54)			
Region degree of contamination	0.28	0.48	0.03	0.561
	(−0.67, 1.24)			
Number of children	−0.44	0.35	−0.06	0.215
	(−1.13, 0.26)			
Perceived severity	−0.36^t^	0.21	−0.08^t^	0.096
	(−0.78, 0.06)			
No access to sports equipment	−0.97	0.71	−0.06	0.172
	(−2.36, 0.42)			
Age	0.01	0.03	0.02	0.762
	(−0.04, 0.06)			
Housing without access to green areas/terrace	0.97	0.75	0.07	0.194
	(−0.50, 2.44)			
Educational attainment	−0.34	0.30	0.06	0.257
	(−0.94, 0.25)			
Perceived stress	0.46	0.58	0.04	0.422
	(−0.67, 1.59)			
Autonomous motivation	0.25	0.31	0.04	0.421
	(−0.36, 0.87)			
Intention	0.85^*^	0.37	0.20^*^	**0.022**
	(0.12, 1.58)			
Gender × intention	0.80^*^	0.39	0.12^*^	**0.041**
	(0.03, 1.57)			
Age × intention	−0.02	0.01	−0.07	0.128
	(−0.05, 0.01)			
Housing without access to green areas/terrace × self-efficacy	0.70^t^	0.38	0.10^t^	0.067
	(−0.05, 1.45)			
Number of children × self-efficacy	0.36^t^	0.19	0.09^t^	0.066
	(−0.02, 0.74)			
Region degree of contamination × intention	0.52^t^	0.30	0.08^t^	0.080
	(−0.06, 1.10)			
Educational attainment × self-efficacy	0.25	0.15	0.08	0.010
	(−0.05, 0.54)			
Part-time job × intention	–1.34^*^	0.66	−0.10^*^	**0.042**
	(−2.63, −0.05)			
Partial unemployment × intention	−0.51	0.64	−0.04	0.425
	(−1.78, 0.75)			
No job × intention	0.14	0.43	0.02	0.744
	(−0.70, 0.98)			
***R*** ^**2**^	**0.44**			
**Adjusted** ***R***^**2**^	**0.39**			

*N = 351. In model 4, dependent variable is minutes of moderate-to-vigorous physical activity per week transformed in squared root; all predictors were mean centered. b, raw coefficient, SE b, standard error of betas, *β*, standardized betas*.

t *p < 0.10*,

* *p < 0.05*,

*** *p < 0.001. Values between parentheses represent confidence intervals. Bold values are significant (p < 0.05)*.

Concerning the moderating role of self-efficacy and intention, the interaction between gender and intention (*β* = 0.12^*^, *p* = 0.041) and the interaction between people having a part-time job and intention (*β* = −0.10^*^, *p* = 0.042) were significantly related to physical activity.

Durbin–Watson test [Durbin and Watson, 1971; Durbin–Watson_Model 4_ = 2.00, quantile–quantile plot (displayed in Supplementary Material)] as well as VIF tests (average VIF_Model 4_ = 1.35) suggested that residuals were normally distributed and not autocorrelated.

**Figure 1:**
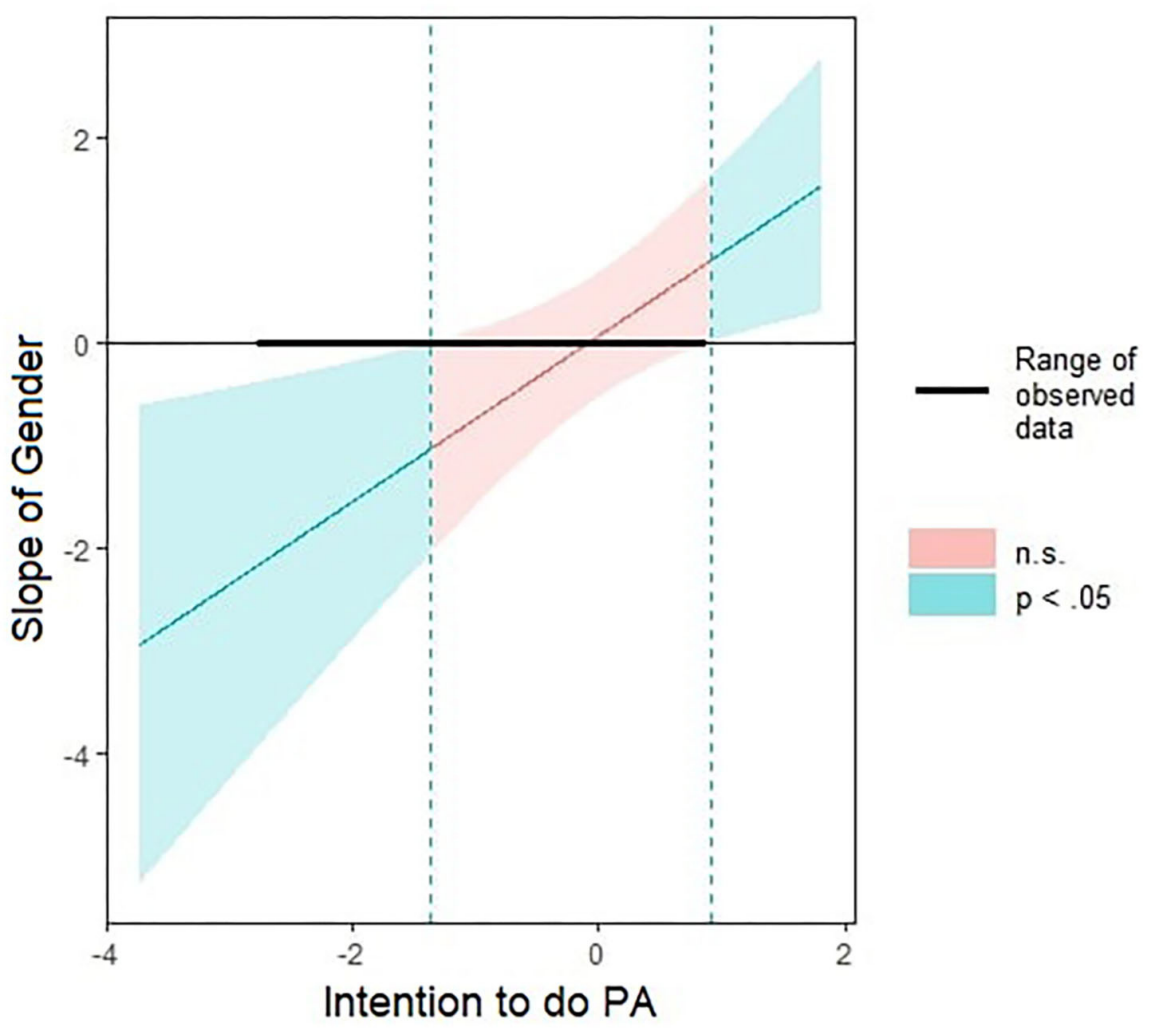
Johnson-Neyman plot of the Interaction Gender x Intention on physical activity. In the x label, Intention standard deviations (SD). In the y level, slope of Gender. Green areas represent significant (*p* < 0.05) slopes, and orange areas represent non-significant slopes. The tick line represents the range of observed data.

**Figure 2:**
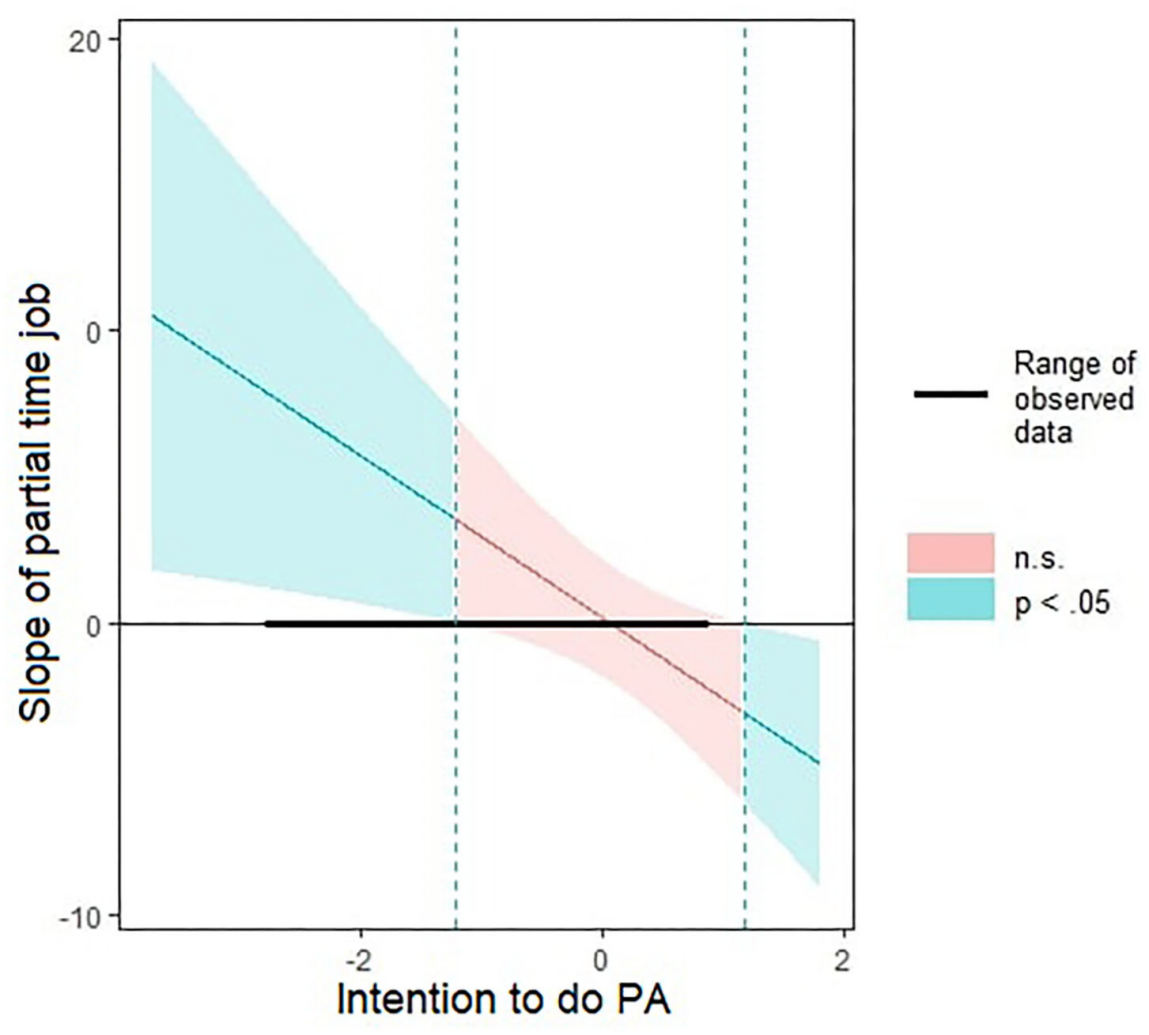
Johnson-Neyman plot of the Interaction Part-time job x Intention on physical activity. In the x label, Intention standard deviations (SD). In the y level, slope of Partial-time job. Green areas represent significant (*p* < 0.05) slopes, and orange areas represent non-significant slopes. The tick line represents the range of observed data.

To simplify simple slopes analyses interpretations, all independent variables were scaled before analyses. All the Johnson Neyman plots are displayed in Supplementary Material. We then decomposed gender × intention and partial-time job × intention interactions using “Interactions” package (Long, 2019) (details of the interactions are displayed in Supplementary Table 2). Intention significantly moderated the association between gender and physical activity (Figure 1). This association was significant when intention was lower or equal to SD = −1.37. In other words, women were more physically active than men when intention was low. Moreover, intention significantly moderated the association between partial-time job and physical activity (Figure 2). This association was significant when intention was inferior to SD = −1.22. In other words, participants with partial-time jobs were less physically active than participants with full-time jobs, but again, only when intention was low.

## Discussion

### Main Findings

Results provide partial support to the hypothesis that individual, sociodemographic, and environmental factors independently predict physical activity (H1). More particularly, we observed a significant role of only one environmental variable (habitat surface area). In contrast, three individual-level variables (usual physical activity, intention, and controlled motivation) significantly predicted physical activity. In other words, people were less physically active when they were little physically active before the COVID-19 lockdown, when they had low intention to be physically active, when they had a high controlled motivation, and when they lived in a small housing.

In contrast, our findings do not provide support to the hypothesis that intention and self-efficacy mediate the association between sociodemographic/environmental factors and physical activity, which contradicts previous studies (Sniehotta et al., 2013; Hagger and Hamilton, 2020). This may be due to the lack of statistical power to carry out mediation analyses.

Finally, intention moderated the association between some sociodemographic variables (i.e., gender and partial-time job) and physical activity, providing some support to H3. More particularly, when intention was low, women and participants with full-time jobs were more physically active than men and participants with partial-time jobs.

### Comparison With Other Studies

The main contribution of this study is to show that individual factors predicted physical activity more than environmental and sociodemographic ones during lockdown, corroborating the results of Rhodes et al. (2020). Although the lockdown has caused sudden changes in people's work, family, and living environment, usual physical activity before the lockdown remained a major predictor of physical activity during this period. This suggests the importance of habits in order to maintain regular physical activity in a suddenly changing environment. Whereas one could have expected external factors to be particularly important in this situation, only one environmental factor (i.e., habitat surface area) significantly predicted physical activity.

At first glance, these results may seem contradictory with several studies showing that the diminution of physical activity during lockdown mostly affected people who were usually physically active (Barkley et al., 2020; Bourdas and Zacharakis, 2020; Castañeda-Babarro et al., 2020; Maltagliati et al., 2020; Meyer et al., 2020; Martínez-de-Quel et al., 2021). Instead, we believe our results nicely complements this line of research. While the lockdown may have negatively affected the evolution of physical activity mostly in usually active individuals, the present study indicates that these individuals were still more active than usually inactive individuals (see also Maltagliati et al., 2020). This suggests that although past physical activity did not completely prevent the damaging impact of lockdown on physical activity, it still had a protective role during lockdown.

The predictive role of intention was in line with past research (e.g., Hagger et al., 2002). In contrast, the lack of significant association between autonomous motivation and physical activity (Teixeira et al., 2012) as well as the association between self-efficacy and physical activity (Hagger et al., 2002) were less expected.

Furthermore, the role of habitat surface area is less studied in the physical activity literature. Some research in leisure-time sitting (Saidj et al., 2015) showed that people living in smaller surfaces tended to spend more hours in a leisure-time sitting. Moreover, habitat surface and characteristics of housing might be an indirect measure of socioeconomic status (Juhn et al., 2011). If we link smaller surfaces with lower socioeconomic status and bigger surfaces with higher socioeconomic status, this could explain our results, as socioeconomic status is related with physical activity (e.g., Ford et al., 1991; Gidlow et al., 2006; Cerin and Leslie, 2008; Beenackers et al., 2012).

Contrary to past research (Cerin and Leslie, 2008; Sniehotta et al., 2013; Hagger and Hamilton, 2020), sociodemographic and environmental effects were not mediated by intention and self-efficacy. COVID-19 has provoked negative impacts on health, employment, and economy in most countries. Nevertheless, recent studies reveal negative impacts are greater for those with lower socioeconomic status (Chung et al., 2020), suggesting that social, health, and economic inequalities are exacerbated due to the epidemic (van Dorn et al., 2020). It seems plausible that the extraordinary challenges of the COVID-19 have revealed a direct association between sociodemographic/environmental and physical activity rather than an association mediated by intention and self-efficacy.

Finally, previous studies have shown that intention and self-efficacy moderate physical activity behaviors (Sniehotta et al., 2013; Schüz et al., 2019; Hagger and Hamilton, 2020); therefore, intention toward physical activity might moderate the effects of sociodemographic and environmental variables on physical activity. For instance, gender and intention toward physical activity have been shown to affect physical activity behaviors in previous work (for a review, see Rhodes and Dickau, 2013).

### Limitations

Measuring physical activity using self-reports was the main limitation of this study, as past research has shown an overestimation of the amount of physical activity when using self-reported physical activity (Dyrstad et al., 2014). In addition, while some methods of power analysis suggest that our study is sufficiently powered to detect mediation (e.g., Schoemann et al., 2017), our methods suggest instead that our study might be insufficiently powered to detected mediation (e.g., Fritz and MacKinnon, 2007). Accordingly, results of our mediation analyses should be interpreted with precaution and need to be replicated in future studies before concluding on the mediating role of intention and self-efficacy in the relationships between sociodemographic/environmental variables and physical activity. Furthermore, because the participants were recruited through social media, our sample was overeducated (i.e., individuals holding a diploma of more than 2 years in France, representing between 14 and 36% of the population; INSEE, 2019) and had fewer children than the average French person (i.e., 0.5 in our sample against the birth rate of 1.87; INSEE, 2019). As such, our results should be interpreted with caution as they are limited to this particular population, which may limit the generalization of our results. In addition, while we observed that individual and environmental/sociodemographic variables independently predicted physical activity, fully disentangling their role is difficult. Indeed, it is possible that usual exercise is determined by sociodemographic (e.g., gender and social status) and environmental factors. As such, some of the environmental/sociodemographic effects may have been partialled out by the inclusion of usual physical activity in the model. Finally, the cross-sectional nature of our study does not allow us to establish causal links. Further longitudinal research during and after the lockdown might allow having more insights about the barriers and levers to physical activity, as well as the mediation and moderation effects of psychological variables.

### Practical Implications

In terms of practical implications, identification of the sociodemographic, environmental, and individual factors of physical activity patterns and levels could benefit physical activity promotion programs. Most countries have implemented two or more lockdowns since the beginning of the pandemic, and the health situation seems to be far from over. Consequently, the promotion of healthy behaviors during lockdowns are critical to preserve mental and physical health, especially for people who have been impacted by unemployment and the economic crisis provoked by the COVID-19 pandemic. Future research should focus on understanding how the health behaviors of individuals from different socioeconomic backgrounds are affected by containment measures in order to better adapt intervention programs.

Broadly speaking, understanding how different levels of factors (i.e., individual, environmental, and sociodemographic) affect physical activity and other health behaviors might give us clues to address social inequalities in physical activity and health (e.g., Hunter et al., 2015). This could be done by targeting either individual-level factors or environmental-level ones. For example, developing intention to be physically active or autonomous forms of motivation for physical activity seems crucial during lockdowns. This may be done by fostering positive attitudes toward physical activity at the individual level or by implementing policies that enable secure, accessible, and child-friendly outdoor places (e.g., public parks) for people living in small and crowded housings. In summary, these findings provide some evidence for the importance of considering multi-level barriers and levers to healthy behavior.

## Data Availability Statement

The datasets presented in this study can be found in online repositories. The names of the repository/repositories and accession number(s) can be found below: https://osf.io/f9c6b/.

## Ethics Statement

The studies involving human participants were reviewed and approved by CERGA Univ. Grenoble Alpes. The patients/participants provided their written informed consent to participate in this study.

## Author Contributions

CT-E analyzed and interpreted the data under the supervision of CF and ACh. CT-E and ACh drafted the manuscript and the remaining authors provided critical revisions. All authors developed the study concept, contributed to the study design and data collection, and approved the final version of this manuscript for submission.

## Conflict of Interest

The authors declare that the research was conducted in the absence of any commercial or financial relationships that could be construed as a potential conflict of interest.
