# Corrigendum: Individual, Sociodemographic, and Environmental Factors Related to Physical Activity During the Spring 2020 COVID-19 Lockdown

**DOI:** 10.3389/fpsyg.2021.816463

**Published:** 2021-12-08

**Authors:** Claudia Teran-Escobar, Cyril Forestier, Clément Ginoux, Sandrine Isoard-Gautheur, Philippe Sarrazin, Anna Clavel, Aïna Chalabaev

**Affiliations:** ^1^Laboratoire SENS, Univ. Grenoble Alpes, Grenoble, France; ^2^Laboratoire PACTE, Univ. Grenoble Alpes, Grenoble, France; ^3^Laboratoire Motricité, Interactions, Performance, MIP - EA4334, Le Mans Université, Le Mans, France

**Keywords:** physical activity, COVID-19 pandemic, psychology, context, exercise

In the original article, the reference for Chen et al. (2020) was incorrectly written as “Chen, P., Mao, L., Nassis, G. P., Harmer, P., Ainsworth, B. E., and Li, F. (2020). Wuhan coronavirus (2019-nCoV): the need to maintain regular physical activity while taking precautions. *J. Sport Health Sci*. 9, 103–104. doi: 10.1016/j.jshs.2020.02.001.” It should be “Chen, P., Mao, L., Nassis, G. P., Harmer, P., Ainsworth, B. E., and Li, F. (2020). Coronavirus disease (COVID-19): The need to maintain regular physical activity while taking precautions. *J. Sport Health Sci*. 9, 103–104. doi: 10.1016/j.jshs.2020.02.001.”

The authors apologize for this error and state that this does not change the scientific conclusions of the article in any way. The original article has been updated.

## Publisher's Note

All claims expressed in this article are solely those of the authors and do not necessarily represent those of their affiliated organizations, or those of the publisher, the editors and the reviewers. Any product that may be evaluated in this article, or claim that may be made by its manufacturer, is not guaranteed or endorsed by the publisher.
