# Supplementary material for: Individual, Sociodemographic, and Environmental Factors Related to Physical Activity During the Spring 2020 COVID-19 Lockdown
Source: Front Psychol. 2021 Mar 16;12:643109. doi: 10.3389/fpsyg.2021.643109 (PMC8008147; doi:10.3389/fpsyg.2021.643109)
Supplement: Supplementary file 1 [file Table_1.DOCX]

Supplementary Material

Supplementary Table S1

Matrix of correlations

| Variable | 1 | 2 | 3 | 4 | 5 | 6 | 7 | 8 | 9 | 10 | 11 | 12 | 13 | 14 | 15 | 16 | 17 | 18 |
| --- | --- | --- | --- | --- | --- | --- | --- | --- | --- | --- | --- | --- | --- | --- | --- | --- | --- | --- |
| 1. MVPA |  |  |  |  |  |  |  |  |  |  |  |  |  |  |  |  |  |  |
| 2. Gend | .12^*^ |  |  |  |  |  |  |  |  |  |  |  |  |  |  |  |  |  |
|  | [.02, .22] |  |  |  |  |  |  |  |  |  |  |  |  |  |  |  |  |  |
| 3. Age | -.07 | .12^*^ |  |  |  |  |  |  |  |  |  |  |  |  |  |  |  |  |
|  | [-.17, .03] | [.02, .22] |  |  |  |  |  |  |  |  |  |  |  |  |  |  |  |  |
| 4. Reg | .01 | .02 | -.06 |  |  |  |  |  |  |  |  |  |  |  |  |  |  |  |
|  | [-.09, .11] | [-.08, .12] | [-.16, .04] |  |  |  |  |  |  |  |  |  |  |  |  |  |  |  |
| 5. Educ | -.05 | -.06 | .26^**^ | .14^**^ |  |  |  |  |  |  |  |  |  |  |  |  |  |  |
|  | [-.15, .05] | [-.16, .04] | [.16, .35] | [.04, .24] |  |  |  |  |  |  |  |  |  |  |  |  |  |  |
| 6. Work | .16^**^ | -.07 | -.26^**^ | -.11^*^ | -.47^**^ |  |  |  |  |  |  |  |  |  |  |  |  |  |
|  | [.06, .26] | [-.17, .03] | [-.35, -.16] | [-.21, -.01] | [-.55, -.39] |  |  |  |  |  |  |  |  |  |  |  |  |  |
| 7. HoType | -.10 | .03 | .01 | .13^**^ | .15^**^ | -.16^**^ |  |  |  |  |  |  |  |  |  |  |  |  |
|  | [-.20, .00] | [-.07, .13] | [-.09, .11] | [.03, .23] | [.05, .25] | [-.25, -.06] |  |  |  |  |  |  |  |  |  |  |  |  |
| 8. Habsurf | .18^**^ | .09 | .05 | -.10 | -.14^**^ | .23^**^ | -.50^**^ |  |  |  |  |  |  |  |  |  |  |  |
|  | [.08, .28] | [-.01, .19] | [-.05, .15] | [-.20, .00] | [-.24, -.04] | [.13, .32] | [-.57, -.42] |  |  |  |  |  |  |  |  |  |  |  |
| 9. NuCh | -.06 | .01 | .17^**^ | -.03 | -.06 | -.04 | -.17^**^ | .25^**^ |  |  |  |  |  |  |  |  |  |  |
|  | [-.16, .04] | [-.09, .11] | [.07, .27] | [-.13, .07] | [-.16, .04] | [-.14, .06] | [-.27, -.07] | [.15, .34] |  |  |  |  |  |  |  |  |  |  |
| 10. SpEq | -.27^**^ | -.02 | .07 | .05 | .01 | -.13^**^ | .14^**^ | -.17^**^ | .03 |  |  |  |  |  |  |  |  |  |
|  | [-.36, -.18] | [-.12, .08] | [-.03, .17] | [-.05, .15] | [-.09, .11] | [-.23, -.03] | [.04, .23] | [-.27, -.07] | [-.08, .13] |  |  |  |  |  |  |  |  |  |
| 11. Media | -.02 | -.00 | -.04 | .06 | .08 | -.04 | .03 | .02 | .00 | .07 |  |  |  |  |  |  |  |  |
|  | [-.12, .08] | [-.10, .10] | [-.14, .06] | [-.04, .16] | [-.02, .18] | [-.14, .06] | [-.07, .13] | [-.08, .12] | [-.10, .10] | [-.03, .17] |  |  |  |  |  |  |  |  |
| 12. Int | .44^**^ | .01 | -.09 | -.02 | -.03 | .03 | -.05 | .03 | -.10 | -.20^**^ | -.02 |  |  |  |  |  |  |  |
|  | [.36, .52] | [-.09, .11] | [-.19, .01] | [-.12, .08] | [-.13, .07] | [-.07, .13] | [-.15, .05] | [-.07, .13] | [-.20, .00] | [-.29, -.10] | [-.12, .08] |  |  |  |  |  |  |  |
| 13. SEf | .47^**^ | .07 | -.04 | -.06 | -.12^*^ | .14^**^ | -.13^*^ | .12^*^ | -.06 | -.26^**^ | -.06 | .71^**^ |  |  |  |  |  |  |
|  | [.39, .55] | [-.03, .17] | [-.14, .06] | [-.16, .04] | [-.22, -.02] | [.04, .24] | [-.23, -.03] | [.02, .22] | [-.16, .04] | [-.35, -.16] | [-.16, .04] | [.65, .75] |  |  |  |  |  |  |
| 14. AMot | .37^**^ | .00 | -.03 | -.09 | -.07 | .06 | -.11^*^ | .11^*^ | -.01 | -.26^**^ | -.06 | .41^**^ | .44^**^ |  |  |  |  |  |
|  | [.28, .45] | [-.10, .10] | [-.13, .07] | [-.19, .01] | [-.16, .04] | [-.04, .16] | [-.21, -.01] | [.01, .21] | [-.11, .09] | [-.35, -.16] | [-.16, .04] | [.32, .49] | [.36, .52] |  |  |  |  |  |
| 15. CMot | -.03 | -.02 | -.28^**^ | .05 | -.02 | .11^*^ | .01 | .01 | -.14^**^ | .04 | .10^*^ | .04 | -.04 | .01 |  |  |  |  |
|  | [-.14, .07] | [-.12, .08] | [-.37, -.18] | [-.05, .15] | [-.12, .08] | [.00, .20] | [-.09, .11] | [-.09, .12] | [-.24, -.04] | [-.06, .14] | [.00, .20] | [-.06, .14] | [-.14, .06] | [-.10, .11] |  |  |  |  |
| 16. SubVi | .28^**^ | .12^*^ | .11^*^ | -.12^*^ | -.04 | .03 | -.12^*^ | .20^**^ | -.05 | -.27^**^ | -.11^*^ | .21^**^ | .39^**^ | .25^**^ | -.03 |  |  |  |
|  | [.19, .37] | [.02, .22] | [.01, .21] | [-.22, -.02] | [-.14, .06] | [-.07, .13] | [-.21, -.02] | [.10, .29] | [-.15, .05] | [-.36, -.18] | [-.21, -.01] | [.11, .30] | [.31, .48] | [.15, .34] | [-.13, .07] |  |  |  |
| 17. Stre | -.02 | .10^*^ | .06 | .01 | -.01 | -.04 | .04 | -.05 | .00 | .06 | -.04 | -.05 | -.03 | .08 | .07 | .09 |  |  |
|  | [-.12, .08] | [.00, .20] | [-.04, .16] | [-.09, .11] | [-.11, .09] | [-.14, .06] | [-.06, .14] | [-.15, .05] | [-.10, .11] | [-.04, .16] | [-.14, .06] | [-.15, .05] | [-.13, .07] | [-.02, .18] | [-.03, .17] | [-.01, .19] |  |  |
| 18. PSe | -.10^*^ | -.04 | .17^**^ | -.04 | -.05 | .04 | .04 | -.01 | .01 | -.02 | .05 | -.02 | -.03 | -.14^**^ | -.09 | -.06 | .08 |  |
|  | [-.20, -.00] | [-.14, .06] | [.07, .26] | [-.13, .06] | [-.15, .05] | [-.06, .14] | [-.06, .14] | [-.11, .09] | [-.09, .11] | [-.12, .08] | [-.05, .15] | [-.12, .08] | [-.13, .07] | [-.24, -.04] | [-.19, .01] | [-.16, .04] | [-.02, .18] |  |
| 19. UsPA | .50^**^ | .16^**^ | -.16^**^ | -.00 | -.17^**^ | .17^**^ | -.19^**^ | .16^**^ | -.02 | -.31^**^ | -.07 | .36^**^ | .42^**^ | .50^**^ | .07 | .22^**^ | -.01 | -.06 |
|  | [.42, .57] | [.07, .26] | [-.25, -.06] | [-.10, .10] | [-.27, -.08] | [.07, .26] | [-.29, -.10] | [.06, .25] | [-.12, .08] | [-.40, -.22] | [-.17, .03] | [.27, .44] | [.33, .50] | [.42, .57] | [-.03, .17] | [.12, .31] | [-.11, .09] | [-.16, .04] |

*Note.* Values in square brackets indicate the 95% confidence interval for each correlation. MVPA = Moderate-to-vigorous physical activity during COVID-19 lockdown, Gender= Gend (1=women, 2=men), , Reg = Region classified in colors (1=Green, least affected zones, 2=Yellow, moderately affected zones, 3=Red, strongly affected zones), Work = employment status (1= full-time job, 2= part-time job, 3 = partial unemployment, 4 =No job), HoType=Housing type (1=Housing with access to green areas/terrace, 2=Housing without access, 3=Housing without classification), Habsurf=Habitat surface area, NuCh=Number of children, SpEq = Access to sports equipment (1=No, 2=Yes), Int=Intention, Sef= Self-efficacy, Amot= Autonomous motivation, Cmot=Controlled motivation, SubVi = Subjective vitality, Stre=Perceived stress, Pse=Perceived severity, UsPA=Usual physical activity before lockdown, Media = Media exposure. ^*^ indicates *p* < .05. ^**^ indicates *p* <

Supplementary Table S2

Summary of Interaction effect between Intention and Gender and, Intention and Part-time job

| **Intention** | | | |
| --- | --- | --- | --- |
| **Variable** | **Focal predictor** | **Intention** | **Interaction** |
|  |  |  |  |
| Gender | .02 | **.20**** | **.12*** |
| No job | .08 | **.20**** | .01 |
| Partial unemployment | .01 | **.20**** | -.04 |
| Part-time job | .01 | **.20**** | **-.10*** |

Note: *N* = 352. Dependent variable is minutes of moderate-to-vigorous physical activity per week transformed in squared root; all predictors were scaled. Focal predictor refers to the effects (standardized beta) of the variable mentioned in the left column on physical activity, ^**^ represents *p* < .01, *** *p* < .001.


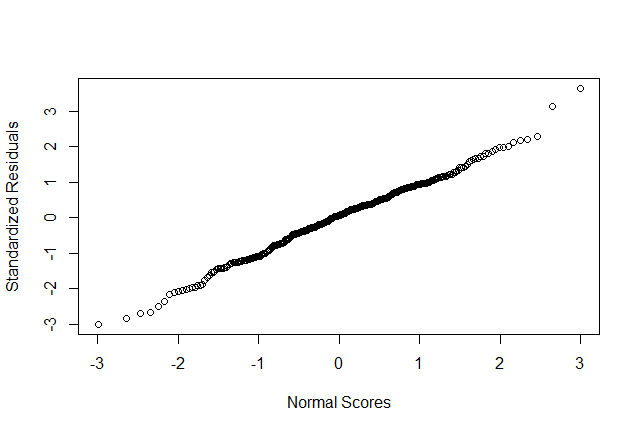


**Supplementary Figure 1***.* Quantile-Quantile plot of standardized residuals of Regression model 1. In the x label, Normal score, in the y label standardized residuals. Each circle represent a residual observation.


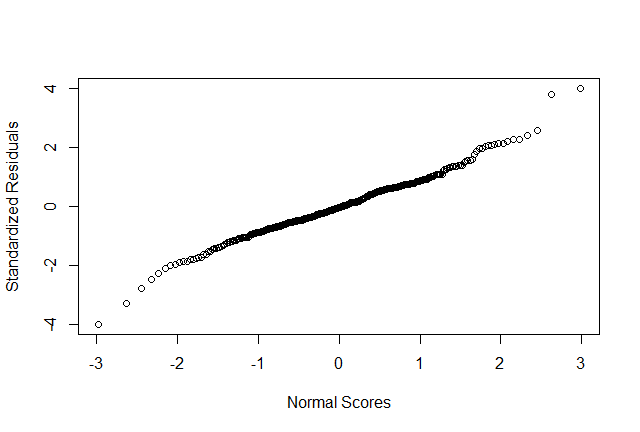


**Supplementary Figure 2.** Quantile-Quantile plot of standardized residuals Regression model 2. In the x label, Normal score, in the y label standardized residuals. Each circle represent a residual observation.

*
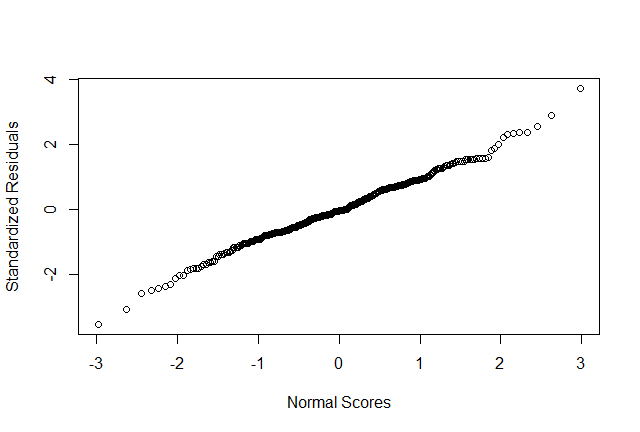
*

**Supplementary Figure 3.** Quantile-Quantile plot of standardized residuals Regression model 3. In the x label, Normal score, in the y label standardized residuals. Each circle represent a residual observation.


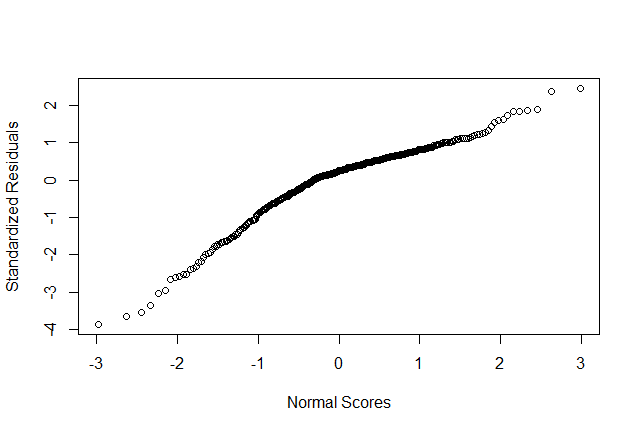


**Supplementary Figure 4.** Quantile-Quantile plot of standardized residuals Regression model 3.1. In the x label, Normal score, in the y label standardized residuals. Each circle represent a residual observation.

*
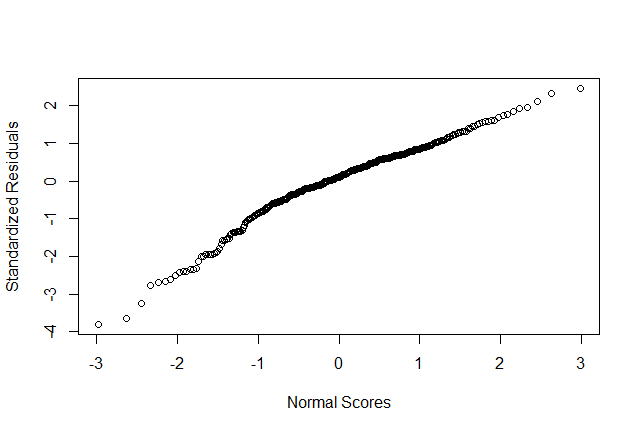
*

**Supplementary Figure 5.** Quantile-Quantile plot of standardized residuals Regression model 3.2. In the x label, Normal score, in the y label standardized residuals. Each circle represent a residual observation.


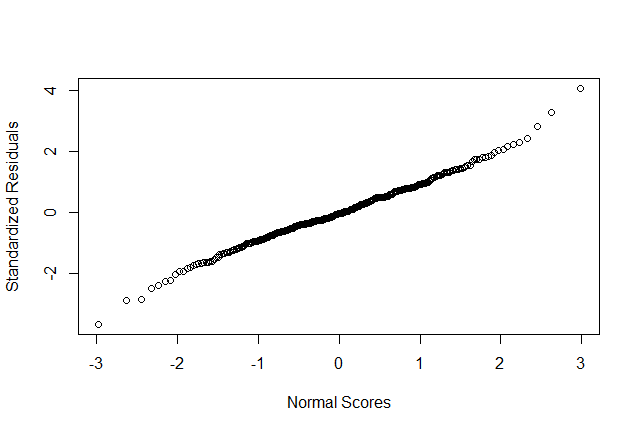


**Supplementary Figure 6.** Quantile-Quantile plot of standardized residuals Regression model 4. In the x label, Normal score, in the y label standardized residuals. Each circle represent a residual observation.

Full scales of physical activity, psychological factors and media exposure

Physical Activity during lockdown

| - Over the past 7 days, indicate the time spent in minutes for each listed type of physical activity… |
| --- |
| - Walking outside |
| - Running outside |
| - Climbing the stairs of the building/house |
| - Doing muscle strengthening exercises (abs, push-ups, squats) or balance / stretching exercises (tai chi, yoga) |
| - Cycling, rowing or doing cardio activities at home |
| - Other physical activities (define which) |

Usual physical activity before the lockdown

| - In general, what did your "profile" look like in terms of physical activity over the past year? If your activity varied greatly from week to week, try to estimate an average | | | |
| --- | --- | --- | --- |
| 1  Sedentary. Being almost completely inactive: reading, TV watching, movies, using computers or doing other sedentary activities during leisure time. | 2  Some physical activity during at least 4 h/week as riding a  bicycle or walking to work, walking or skiing with the family, gardening, fishing, table tennis, bowling, etc. | 3  Regular physical activity and training (moderate PA) such as heavy gardening, running, swimming, calisthenics, tennis, badminton and similar activities for at least 2–3 h/week. | 4  Regular hard physical training for competition sports  (vigorous PA): running events, orienteering, skiing, swimming, soccer, racing, European handboll, etc. Several times per week. |

Intention

| - Since lockdown, to what extent do you intend to do 30 minutes of moderate to vigorous physical activity at least 5 days a week, as recommended by health authorities? | | | | | | |
| --- | --- | --- | --- | --- | --- | --- |
| 1  No intention at all | 2 | 3 | 4 | 5 | 6 | 7  Totally the intention |

Self-efficacy

| - Since lockdown, how confident are you in your ability to do 30 minutes of moderate to vigorous physical activity at least 5 days a week as recommended by health authorities? | | | | | | |
| --- | --- | --- | --- | --- | --- | --- |
| 1  No confident at all | 2 | 3 | 4 | 5 | 6 | 7  Totally confident |

Autonomous Motivation

| - We would like to know your motivations when you do physical activities, in other words, why you do physical activity or exercise. Indicate the extent to which each of the following statements currently corresponds to one of your reasons for being physically active. - For the pleasure I experience when I practice PA | | | | | | |
| --- | --- | --- | --- | --- | --- | --- |
| 1  (Does not match at all | 2 | 3 | 4 | 5 | 6 | 7  Matches very strongly |
| - Because personally, I consider it as a factor of well-being. | | | | | | |
| 1  (Does not match at all | 2 | 3 | 4 | 5 | 6 | 7  Matches very strongly |
| - For the pleasant feelings that PA provides me. | | | | | | |
| 1  (Does not match at all | 2 | 3 | 4 | 5 | 6 | 7  Matches very strongly |
| - Because I believe that PA is a good thing for my personal growth. | | | | | | |
| 1  (Does not match at all | 2 | 3 | 4 | 5 | 6 | 7  Matches very strongly |

Controlled motivation

| - We would like to know your motivations when you do physical activities, in other words, why you do physical activity or exercise. Indicate the extent to which each of the following statements currently corresponds to one of your reasons for being physically active. - Because I would feel ashamed if I wasn't physically active. | | | | | | |
| --- | --- | --- | --- | --- | --- | --- |
| 1  (Does not match at all | 2 | 3 | 4 | 5 | 6 | 7  Matches very strongly |
| - Because some people around pressure me to do it | | | | | | |
| 1  (Does not match at all | 2 | 3 | 4 | 5 | 6 | 7  Matches very strongly |
| - In order not to hear the criticisms of certain people. | | | | | | |
| 1  (Does not match at all | 2 | 3 | 4 | 5 | 6 | 7  Matches very strongly |
| - Because I would feel bad if I did not make this effort. (REMOVED) | | | | | | |
| 1  (Does not match at all | 2 | 3 | 4 | 5 | 6 | 7  Matches very strongly |

Subjective vitality

| - In the last seven days... - I felt alive and vital | | | | | | |
| --- | --- | --- | --- | --- | --- | --- |
| 1  Completely disagree) | 2 | 3 | 4 | 5 | 6 | 7  Completely agree |
| - I have energy and spirit | | | | | | |
| 1  Completely disagree) | 2 | 3 | 4 | 5 | 6 | 7  Completely agree |
| - I look forward to each new day | | | | | | |
| 1  Completely disagree) | 2 | 3 | 4 | 5 | 6 | 7  Completely agree |
| - I nearly always feel alert and awake | | | | | | |
| 1  Completely disagree) | 2 | 3 | 4 | 5 | 6 | 7  Completely agree |
| - I feel energized | | | | | | |
| 1  Completely disagree) | 2 | 3 | 4 | 5 | 6 | 7  Completely agree |

Perceived stress

| - In the last week… - How often have you felt you were unable to control the important things in your life? | | | | | | |
| --- | --- | --- | --- | --- | --- | --- |
| 1  Never | 2 | 3 | 4 | 5 | 6 | 7  Always |
| - How often have you felt confident about your ability to handle your | | | | | | |
| 1  Never | 2 | 3 | 4 | 5 | 6 | 7  Always |
| - How often have you felt difficulties were piling up so high that you could not overcome them? | | | | | | |
| 1  Never | 2 | 3 | 4 | 5 | 6 | 7  Always |
| - How often have you felt that things were going your way? | | | | | | |
| 1  Never | 2 | 3 | 4 | 5 | 6 | 7  Always |

Perceived risks

Perceived susceptibility

| - I have an increased risk of falling ill with coronavirus disease | | | | | | |
| --- | --- | --- | --- | --- | --- | --- |
| 1  Completely disagree) | 2 | 3 | 4 | 5 | 6 | 7  Completely agree |
| - I am concerned about the risk of falling ill with coronavirus disease | | | | | | |
| 1  Completely disagree) | 2 | 3 | 4 | 5 | 6 | 7  Completely agree |
| - I get sick more easily than other people my age | | | | | | |
| 1  Completely disagree) | 2 | 3 | 4 | 5 | 6 | 7  Completely agree |

Perceived severity

| - I have an increased risk of falling ill with coronavirus disease | | | | | | |
| --- | --- | --- | --- | --- | --- | --- |
| 1  Completely disagree) | 2 | 3 | 4 | 5 | 6 | 7  Completely agree |
| - I am afraid the coronavirus disease will make me very sick | | | | | | |
| 1  Completely disagree) | 2 | 3 | 4 | 5 | 6 | 7  Completely agree |
| - I cannot stand the coronavirus disease because of my general health | | | | | | |
| 1  Completely disagree) | 2 | 3 | 4 | 5 | 6 | 7  Completely agree |

Media Exposure

| - The following question is intended to help you understand your key sources of information since the beginning of the pandemic. For each source of information below, indicate whether your consultations are decreasing or increasing, on a scale from 1 to 10. - Television | | | | | | | | | |
| --- | --- | --- | --- | --- | --- | --- | --- | --- | --- |
| 1  Decrease of informations | 2 | 3 | 4 | 5  Constancy | 6 | 7 | 8 | 9 | 10  Increase of information |
| - Internet | | | | | | | | | |
| 1  Decrease of informations | 2 | 3 | 4 | 5  Constancy | 6 | 7 | 8 | 9 | 10  Increase of information |
| - Social media | | | | | | | | | |
| 1  Decrease of informations | 2 | 3 | 4 | 5  Constancy | 6 | 7 | 8 | 9 | 10  Increase of information |
| - Press | | | | | | | | | |
| 1  Decrease of informations | 2 | 3 | 4 | 5  Constancy | 6 | 7 | 8 | 9 | 10  Increase of information |
